# Personality traits are directly associated with anti-black prejudice in the United States

**DOI:** 10.1371/journal.pone.0235436

**Published:** 2020-07-01

**Authors:** Chujun Lin, R. Michael Alvarez

**Affiliations:** Division of Humanities and Social Sciences, California Institute of Technology, Pasadena, California, United States of America; Sogang University (South Korea), REPUBLIC OF KOREA

## Abstract

Modern psychological theories postulate that individual differences in prejudice are determined by social and ideological attitudes instead of personality. For example, the dual-process motivational (DPM) model argues that personality does not directly associate with prejudice when controlling for the attitudinal variables that capture the *authoritarian-conservatism motivation* and the *dominance motivation*. Previous studies testing the DPM model largely relied on convenience samples and/or European samples, and have produced inconsistent results. Here we examined the extent to which anti-black prejudice was associated with the Big Five personality traits and social and ideological attitudes (authoritarianism, social dominance orientation, political party affiliation) in two large probability samples of the general population (*N*_1_ = 3,132; *N*_2_ = 2,483) from the American National Election Studies (ANES). We performed structural equation modeling (SEM) to test the causal assumptions between the latent variables and used survey weights to generate estimates that were representative of the population. Different from prior theories, across both datasets we found that two personality traits, agreeableness and conscientiousness, were directly associated with anti-black prejudice when controlling for authoritarianism, social dominance orientation, and political party affiliation. We also found that a substantial part of the associations between personality traits and anti-black prejudice were mediated through those social and ideological attitudes, which might serve as candidates for prejudice-reduction interventions in the real world.

## Introduction

Prejudice has been a critical problem throughout the world, with recent examples ranging from racial profiling and gender wage gaps in the United States [1,2], to anti-immigrant attitudes in Europe [3]. Understanding the sources of prejudice is an important goal of much research across social psychology, political science, and neuroscience [4–8].

A key question is to what extent individual differences in prejudice are driven by differences in social and ideological attitudes, which are relatively changeable, and differences in personality traits, which are relatively stable features of individuals. Answering this question is essential both theoretically for the conceptualization of prejudice (e.g., as an entirely flexible attitude, or a partially flexible and partially stable variable) and practically for the reduction of prejudice (e.g., what variables should educators and policy makers aim to intervene and how much of an effect to expect).

The dual-process motivational model (DPM) of prejudice [9] is a pioneering framework that formally conceptualized and tested this question. The DPM model postulates that prejudiced intergroup attitudes are driven by two distinct motivations, a threat-induced *authoritarian-conservatism motivation* that expresses attitudes of pursuing social control, security, and conformity, and opposing autonomy and individual freedom (which could be measured with the right-wing authoritarianism scale [RWA]), and a competition-induced *dominance motivation* that expresses attitudes of pursuing dominance, power, and superiority over others, and opposing egalitarian and altruistic social concerns for others (which could be measured with the social dominance orientation scale [SDO]); and that personality traits are not directly associated with prejudice, but have substantial impacts on those attitudes. These causal assumptions were supported with findings from six student samples in the original study [9].

However, the reproducibility and generalizability of results from student samples are debated [10–12] due to their skewed distributions in features such as age and education that might bias the estimation of the relationship between personality and attitudes [13–15; but see [15]]. Subsequent studies testing the DPM model using more diverse samples have produced inconsistent results (Table 1; our search of literature was restricted to studies that included all relevant measures, namely, the Big Five personality traits, RWA, SDO, and prejudice, and that were published in English). Four samples—all being students or parents and adult friends of the students—indicated that none of the Big Five personality traits had any direct association with prejudice once RWA and SDO were controlled for; whereas, the other samples—two from the general public and one being a large meta-analytic sample—showed direct associations between personality traits and prejudice (Table 1). These discrepant findings highlight the importance of elucidating the relationship between personality and prejudice in larger and more representative samples.

**Table 1:** Overview of literature that directly tested the relationships between the Big Five personality traits, RWA, SDO, and prejudice.

Source	Sample Characteristics	Prejudice Metric	Analysis Method	Direct Association of Personality (beyond RWA and SDO)
Cohrs, Kämpfe-Hargrave, & Riemann, 2012 (Study 1)	N = 193 individuals from the general population of Germany (125 females, 64 males, and 4 other; Age (Range = [18, 67], M = 34, SD = 12))	Generalized prejudice	SEM with maximum likelihood estimation	Yes. Agreeableness.
Cohrs, Kämpfe-Hargrave, & Riemann, 2012 (Study 2)	N = 424 individuals from the Jena Twin Registry in Germany, one individual was selected from each pair of twins (321 females and 103 males; Age (Range = [18, 82], M = 34, SD = 13))	Generalized prejudice	SEM with maximum likelihood estimation	Yes. Agreeableness and Openness to Experience.
Duriez & Soenens, 2006	N = 328 first year psychology students from a university in Belgium (80% females; Age (Range = [18, 24], M = 18.5))	Racial Prejudice	SEM with maximum likelihood estimation	No
Ekehammar, Akrami, Gylje, & Zakrisson, 2004	N = 183 students from a university in Sweden (97 females and 86 males; Age (M = 23))	Generalized prejudice	SEM with maximum likelihood estimation	No
Hodson, Hogg, & MacInnis, 2009	N = 197 undergraduate students from a university in Canada (156 females and 41 males; Age (Range = [17, 39], M = 20, SD = 2.5))	Modern racial prejudice (towards immigrants)	SEM with maximum likelihood estimation	No
McFarland, 2010 (Study 3)	N = 200 adults (mostly parents or other nonstudent adults known by the students who participated in the author’s other studies) from the United States (111 females and 89 males; Age (M = 43))	Generalized prejudice	Linear regression	No
Sibley & Duckitt, 2008	N = 2,479 participants across nine studies (students/adolescents in 67% of the studies and adults in 33% of the studies; participants were from Europe in 90% of the studies and from United States in 10% of the studies)	Of the included studies: Racism (70%), Generalized prejudice (20%), Sexism (10%).	Meta-analysis of Bivariate and Partial Correlations	Yes. Agreeableness.

Here we investigated the relationships between the Big Five personality traits, social and ideological attitudes, and anti-black prejudice in two large probability samples (*N*_1_ = 3,132; *N*_2_ = 2,483) of the white population in the United States—a country with longstanding and prominent issues with prejudice, especially the racial prejudice of whites against blacks [6,16,17]. We carried out our investigation within the context of two presidential elections that featured heightened tensions between blacks and whites—the 2012 election where the first black president was reelected and the 2016 election where a president embracing racist rhetoric was elected, using the 2012 and 2016 ANES datasets collected immediately following the elections. Besides the attitudinal variables measuring the *authoritarian-conservatism motivation* and the *dominance motivation* proposed in the DPM model, we included measures of political party affiliation, considering the nature of the American polarizing party system and its relations to personality and social attitudes [18–22]. We hypothesized that three personality traits, agreeableness, conscientiousness, and openness to experience, would be directly associated with anti-black prejudice beyond social and ideological attitudes. To account for measurement error and to uncover the underlying relationships between these variables, we performed structural equation modeling (SEM). To generate estimates that could be generalized to the U.S. white adult population and thus informative for policy makers, we applied survey weights to all analyses, which accounted for the probability of household selection, respondent selection, nonresponse, and random sampling error [23,24]. All data and analysis codes can be accessed at Open Science Framework https://osf.io/zhtvf/?view_only=134010a7a05e4d0ab8cbd6b2927f98eb.

### The relationship between personality and prejudice

The hypothesis that individual differences in prejudice are inherent features of individuals was motivated by the empirical finding that people who are prejudiced against one group also tend to be prejudiced against other groups [25–27]. This suggests that prejudice might be rooted in one’s personality. Adorno, Frenkel-Brunswik, Levinson, and Sanford (1950) initially proposed that generalized prejudice was an expression of the authoritarian personality, which describes an individual’s commitment to social norms and compliance to authority and can be measured with the RWA scale [26]. Subsequent research found that SDO, which was regarded as a personality variable and describes an individual’s preference for a hierarchical versus equal intergroup relation, also powerfully predicted prejudice against various groups [28,29]. However, recent research, including the DPM model, has proposed that RWA and SDO are measures of more transient attitudes instead of more stable personality [9,30]. The conceptualization of RWA and SDO as attitudinal or personality variables remains debated.

The development of a more reliable framework of personality, namely, the Big Five [31,32], has advanced the investigation on the relationship between personality and prejudice. The Big Five (extraversion, agreeableness, conscientiousness, emotional stability, and openness to experience) has been demonstrated by a large body of research to explain most individual differences in personality [33–35]. Individuals with higher scores on agreeableness have been shown to be more likely to help victims [36,37], suppress negative reactions to traditional targets of prejudice [38–40], and evaluate contacts with blacks more favorably [41]. The association between agreeableness and prejudice was found to be mediated by SDO [42]. Conscientiousness describes the individual differences in impulse control, determination to achieve, and preference for conformity and security [43,44]; individuals with higher scores on contentiousness have been shown to be more risk averse [45], more likely to hold group-centric policy positions [18], and less likely to hold positive attitudes towards immigrants or minority rights [46–48]. Individuals with higher scores on openness to experience have been shown to be less sensitive to social threat [49,50], express more tolerant racial attitudes towards blacks [51], and tend to initiate contacts with blacks and interpret those contacts favorably [41]. The association between openness to experience and prejudice was found to be mediated by RWA [42]. Extraversion and emotional stability are generally found to be uncorrelated with prejudiced attitudes [42].

Notably, prior research testing the causal assumptions between the Big Five personality traits and prejudice based on the DPM framework was largely built on findings from European samples (Table 1). Given that cultural, social, and political contexts have been shown to powerfully shape personality and social attitudes [13,42], our present investigation with large representative samples from the United States will provide new insights into their relationship.

### Anti-black prejudice in the United States

Racial tensions between blacks and whites have played a significant role in U.S. politics and society since the nation’s founding. Contemporary reports about a white person falsely calling the police on a black person, a white police officer mistakenly shooting a black man, or white students harassing or bullying black students, are no stranger to everyday news. Although the passage of important federal legislation has eliminated many forms of economic and political discrimination against blacks, and some studies argued that direct forms of racial prejudice have become less apparent [52], others have shown that fewer self-reports of overt racism in recent years does not necessarily mean that white individuals in the U.S. no longer possess prejudicial opinions towards blacks [53].

A considerable amount of research has demonstrated that anti-black prejudice remains a strong factor in political decision making for many white Americans in the 2008 and 2012 elections and during Obama’s presidency [54–57]. Recent studies examining the Trump campaign and the 2016 election have shown that animus towards non-whites and immigrants was largely revealed in the election [58,59]. It’s thus likely that the 2012 and 2016 elections stimulated many voters to consider the issue of race and ethnicity. It was within the context of the enduring tensions between blacks and whites in the U.S. and the two critical periods of presidential elections that our present research utilized the two ANES datasets and focused on white respondents to understand the relationship between personality and anti-black prejudice in the United States.

## General methods

### Participants

This project meets the criteria for exemption from the Institutional Review Board of the California Institute of Technology. The 2012 ANES Time Series Study (most up-to-date version: May 24, 2016) collected data from two distinct and independently drawn probability samples: the face-to-face interview sample (*N* = 1,929) and the internet survey sample (*N* = 3,581). The sample universe for the face-to-face interviews included all U.S. adult citizens in the U.S. postal service address system from the 48 contiguous states (Alaska and Hawaii were excluded) and the District of Columbia; address-based sampling allowed access to an estimated 98% of U.S. households. The sample universe for the internet surveys included all U.S. adult citizens in the U.S. postal service address system as well as the U.S. residential landline telephone system; random-digit dialing sampling allowed access to an estimated 75% of U.S. households.

In our present research, we utilized a subset of the 2012 ANES Time Series Study data that were collected within two and a half months following the presidential election and were from participants who self-identified as “White non-Hispanic” (59%). Participants with missing data (e.g., “Refused”, “Don’t know”, “Not asked”) for any of the relevant measures listed below were excluded (*n* = 377). After exclusion, our 2012 ANES dataset consisted of responses from 3,132 white, non-Hispanic participants (1,550 females and 1,582 males, Age (*Range* = [18, 90], *M* = 51.89, *SD* = 16.53)).

The 2016 ANES Time Series Study (most up-to-date version: Sep 4, 2019) also collected data from two distinct and independently drawn probability samples: the face-to-face interview sample (*N* = 1,180) and the internet survey sample (*N* = 3,090). The sample universe for the face-to-face interviews included 222.6 million U.S. adult citizens in the U.S. postal service address system from the 48 contiguous states and the District of Columbia. The sample universe for the internet surveys included 224.1 million U.S. adult citizens in the U.S. postal service address system from the 50 states and the District of Columbia.

In our present research, we utilized a subset of the 2016 ANES Time Series Study data that were collected within two months following the presidential election and were from participants who self-identified as “White non-Hispanic” (71%). After excluding participants with missing data for any relevant measures (*n* = 555), our 2016 ANES dataset consisted of responses from 2,483 white, non-Hispanic participants (1,300 females and 1,165 males, others refused; Age (*Range* = [18, 90], *M* = 50.95, *SD* = 17.56)).

### Survey weights

The 2012 and 2016 ANES Time Series Study provided survey weights for generating estimates that were representative of the target populations [23,24] and thus informative for policy makers in the real world. Survey weights were post-stratified quantities assigned to each observation in the dataset that adjusted the estimates so that they matched known population proportions for certain characteristics. In ANES, these characteristics included important demographic features that were known to be correlated with personality and social attitudes, such as age, gender, educational attainment, income, and marital status. “Each weight accounts for the probability of household selection, the probability of respondent selection within the household, nonresponse, and random sampling error” [23]. The use of survey weights should also help alleviate potential biases produced by mode differences (face-to-face interviews versus internet surveys) in our datasets [60]. We applied survey weights for computing descriptive statistics of all measures and for generating estimates in all models.

### Measures

#### Anti-black prejudice

Three different measures were included to assess anti-black prejudice: negative black affect, negative stereotypes, and symbolic racism, following the recommendations of previous research [54,55]. Firstly, negative black affect was assessed with a 101-point (from 0 to 100) warm/cold feeling thermometer towards blacks in both the 2012 and 2016 ANES; in addition, two 5-point Likert-scale items evaluating sympathy and admiration for blacks were also included to assess negative black affect in the 2012 ANES. Secondly, negative stereotypes were assessed with two 5-point Likert-scale items evaluating stereotypes of blacks’ work ethic as well as intelligence (in the 2012 ANES) or violence (in the 2016 ANES). To control for potential idiosyncratic differences in the affect and stereotypes different participants might have towards people in general (for example, a low feeling thermometer towards blacks might indicate aversion against blacks specifically or a pessimistic view of people in general), we subtracted a participant’s score given to blacks from his/her score given to whites [17,55,61]. Thirdly, symbolic racism was assessed with four 5-point Likert-scale items asking whether “blacks should work their way up without special favors”, “past slavery made it more difficult for blacks”, “blacks had gotten less than deserved”, and “blacks must try harder to get ahead” in both the 2012 and 2016 ANES. All items across the three different measures were first scaled to the unit interval and then averaged (reverse coded if needed) to create a composite measure of anti-black prejudice (see Table 2 for weighted descriptive statistics).

**Table 2:** Descriptive statistics of all relevant measures in the 2012 and 2016 ANES datasets calculated with applying survey weights.

	**E**	**A**	**C**	**ES**	**O**	**AUT**	**SDO**	**PAR**	**Prejudice**
2012 ANES									
E	--								
A	0.01	--							
C	0.11***	0.20***	--						
ES	0.11***	0.32***	0.31***	--					
O	0.33***	0.14***	0.24***	0.25***	--				
AUT	0.01	-0.02	0.01	-0.08***	-0.14***	--			
SDO	-0.02	-0.11***	0.05**	0.06**	-0.13***	0.17***	--		
PAR	0.00	-0.04	0.07***	0.05**	-0.11***	0.24***	0.51***		
Prejudice	0.00	-0.10***	0.09***	-0.01	-0.08***	0.34***	0.47***	0.40***	--
Mean	0.52	0.69	0.78	0.65	0.63	0.38	0.35	0.52	0.61
SD	0.22	0.18	0.18	0.21	0.19	0.20	0.17	0.26	0.15
Alpha	0.58	0.38	0.51	0.64	0.43	0.59	0.79	0.85	0.81
2016 ANAES									
E	--								
A	-0.07**	--							
C	0.12***	0.29***	--						
ES	0.05*	0.30***	0.37***	--					
O	0.28***	0.18***	0.22***	0.19***	--				
AUT	-0.00	-0.04	0.03	-0.06**	-0.22***	--			
SDO	-0.01	-0.12***	-0.01	0.03	-0.25***	0.31***	--		
PAR	0.00	-0.01	0.09***	0.06**	-0.19***	0.35***	0.50***		
Prejudice	0.05*	-0.05*	0.14***	0.02	-0.18***	0.49***	0.52***	0.54***	--
Mean	0.54	0.70	0.78	0.65	0.66	0.35	0.29	0.52	0.57
SD	0.24	0.19	0.18	0.21	0.19	0.21	0.17	0.27	0.19
Alpha	0.58	0.38	0.55	0.57	0.44	0.63	0.70	0.83	0.83

The weighted Pearson correlations were estimated using bootstrapping (1000 iterations), with bootstrapped standard errors to account for potential heteroscedasticity. E = extraversion, A = agreeableness, C = conscientiousness, ES = emotional stability, O = openness to experience, AUT = authoritarianism, PAR = political party affiliation (higher scores for closer affiliation with the Republican party), Prejudice = anti-black prejudice. Significance code: *** p < 0.001, ** p < 0.01, * p < 0.05.

#### Authoritarianism

Authoritarianism was included in our model to capture the individual differences in the *authoritarian-conservatism motivation* proposed in the DPM model, and was assessed using four pairs of childrearing values [62,63] in both the 2012 and 2016 ANES. Prior research has demonstrated that childrearing values are reliable indicators of authoritarianism [30,64]. Participants indicated desirable qualities in children by choosing between independence versus respect for elders, curiosity versus good manners, self-reliance versus obedience, and being considerate versus well behaved. Item responses were averaged (reverse coded if needed) to create a composite measure of authoritarianism, which was further scaled to the unit interval (Table 2).

#### SDO

SDO was included in our model to capture the individual differences in the *dominance motivation* proposed in the DPM model. Six 5-point Likert-scale items modified from the SDO scale [29] were used to measure an individual’s social dominance orientation in the 2012 ANES. Questions concerned whether “society should make sure everyone has an equal opportunity”, “we would be better off if worry less about equality”, “it was not a big problem if some had more chance”, “we would have fewer problems if people were treated more fairly”, “it was a big problem that we didn’t give everyone an equal chance”, and “we had gone too far in pushing equal rights”. The first four of the six items were used to measure SDO in the 2016 ANES. Item responses were averaged (reverse coded if needed) to create a composite measure of SDO, which was further scaled to the unit interval (Table 2).

#### Political party affiliation

Measures of how strongly an individual was associated with the Democratic or Republican parties were included in our model to capture potential effects of American polarizing party system on personality and social attitudes, and were assessed with three items in both the 2012 and 2016 ANES: a 7-point Likert-scale item explicitly asking for participants’ party identification, and two 101-point (from 0 to 100) warm/cold feeling thermometer towards the Democratic party and the Republican party. Item scores were first scaled to the unit interval and then averaged (reverse coded if needed) to create a composite measure of political party affiliation (Table 2).

#### The Big Five personality traits

The Ten Item Personality Inventory (TIPI) was used to assess the Big Five personality traits [65] in both the 2012 and 2016 ANES. The validity of this short inventory has been confirmed by prior research [66–68]. Each personality trait was measured with two items (one positive, one negative) and each item consisted of two descriptors (e.g., “extraverted, enthusiastic”). For each item, participants were asked to rate the extent to which the pair of descriptors applied to them on a 7-point Likert scale. The two item responses for each personality trait were averaged (reverse coded if needed) to create a composite measure of the personality trait (Table 2).

### Measurement model

We performed structural equation modeling with latent variables. Each latent variable was linked to one composite indicator—that is, the composite indicators of the five personality traits, authoritarianism, SDO, party affiliation, and anti-black prejudice. Following previous research [69,70], we adjusted for potential measurement error by setting the error variance of the composite indicator to one minus the reliability (Cronbach’s *α*) times the variance of the composite indicator, both of which were computed with applying survey weights, (1 − *α*_*weighted*_) × *VAR*_*weighted*_. This adjustment of measurement errors allowed an analysis of the relationship among the latent variables rather than the composite measures in the structural equation models.

### Structural equation modeling

**Fig 1:**
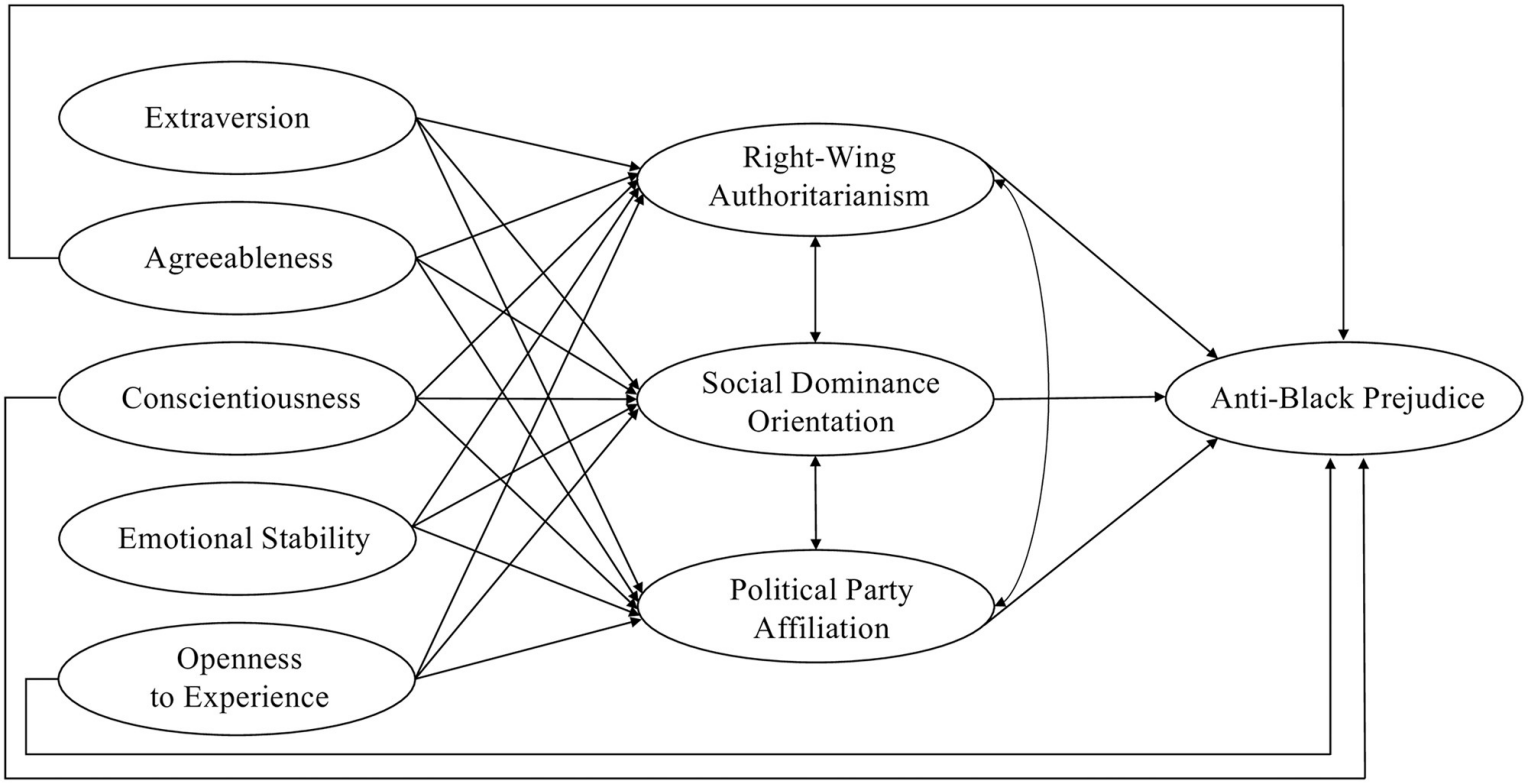
Hypothesized latent structural relationships between the Big Five personality traits, authoritarianism, SDO, political party affiliation, and anti-black prejudice. All the Big Five personality traits were allowed to correlate; to simplify the diagram, these correlations, as well as the composite indicators and the paths from the composite indicators to the latent variables were not depicted in the graph.

Structural equation modeling allows for testing of causal assumptions between the latent variables, with significant findings lending credibility to the causal assumptions (but not proving their validity). We hypothesized that three of the Big Five personality traits, agreeableness, conscientiousness, and openness to experience, would have significant direct associations with anti-black prejudice when controlling for authoritarianism, SDO, and party affiliation (Fig 1). We compared our hypothesized model with two alternative models recommended by Ekehammar, Akrami, Gylje, and Zakrisson (2004). One alternative model challenged the prevailing implicit assumption that individual differences in social and ideological attitudes cause individual differences in prejudice, and instead assumed that anti-black prejudice was the causal determinant of authoritarianism, SDO, and party affiliation (S1 Appendix (I)). The other alternative model challenged the prevailing implicit assumption that individual differences in personality traits cause individual differences in social and ideological attitudes, and instead assumed that authoritarianism, SDO, and party affiliation predisposed the individual differences in personality, which in turn influenced anti-black prejudice (S1 Appendix (II)). We also reported results of a conceptual replication of the DPM model (S2 Appendix; only two attitudinal variables that capture the *authoritarian-conservatism motivation* and the *dominance motivation* were included in the model, without controlling for political party affiliation) and results of our hypothesized model tested using unweighted data (S3 Appendix).

The structural equation models were first estimated with maximum likelihood using the R package *lavaan*, and then refitted to the population covariance matrix (computed from the R package *survey*) using the R package *lavaan*.*survey*, in order to generate estimates for the target population. As recommended in previous studies [71,72], model fit was assessed using multiple indices: root-mean-square error of approximation (RMSEA close to or below 0.06 indicates good fit), standardized root-mean-square residual (SRMR close to or below 0.08 indicates good fit), comparative fit index and adjusted goodness-of-fit index (CFI and AGFI close to or above 0.95 indicate good fit), and normed Chi-square (NC = χ^2^/*df*, NC between 1.0 to 5.0 indicates acceptable fit and NC close to or below 2.0 indicates good fit).

## Results

To elucidate the relationships between our latent variables in detail, analyses of our hypothesized structural equation model were carried out in three separate steps (Fig 1). The first step provided a preliminary estimation of the relationship between the three hypothesized personality traits and anti-black prejudice when social and ideological attitudes were not controlled for—that is, only the direct paths from agreeableness, conscientiousness, and openness to experience to anti-black prejudice were included. We found significant associations of all three personality traits with anti-black prejudice across both datasets (*β*_A_ = -.31, *β*_C_ = .43, *β*_O_ = -.28 in the 2012 ANES; *β*_A_ = -.37, *β*_C_ = .71, *β*_O_ = -.59 in the 2016 ANES). However, the model fit was unacceptable (χ^2^/*df* = 75.39 in the 2012 ANES and 92.99 in the 2016 ANES).

The second step illustrated how social and ideological attitudes mediated the associations between personality traits and anti-black prejudice—that is, all hypothesized paths except the three directly linking personality traits and anti-black prejudice were included. The associations between the Big Five personality traits and the social and ideological attitudes found in the two datasets were slightly different, but the directions of all associations were consistent across datasets. In the 2012 ANES dataset, all four personality traits other than agreeableness were significantly associated with authoritarianism (*β*_E_ = .42, *β*_C_ = .44, *β*_ES_ = -.15, *β*_O_ = -.90); all five personality traits were significantly associated with SDO (*β*_E_ = .20, *β*_A_ = -.52, *β*_C_ = .36, *β*_ES_ = .35, *β*_O_ = -.70) as well as party affiliation (*β*_E_ = .43, *β*_A_ = -.31, *β*_C_ = .57, *β*_ES_ = .32, *β*_O_ = -1.15). In the 2016 ANES dataset, all five personality traits were significantly associated with authoritarianism (*β*_E_ = .53, *β*_A_ = .60, *β*_C_ = .35, *β*_ES_ = -.25, *β*_O_ = -1.27), all four personality traits other than agreeableness were significantly associated with SDO (*β*_E_ = .25, *β*_C_ = .26, *β*_ES_ = .24, *β*_O_ = -.85), and three personality traits were significantly associated with party affiliation (*β*_E_ = .55, *β*_C_ = .40, *β*_O_ = -1.46). As expected, authoritarianism, SDO, and party affiliation showed significant positive associations with anti-black prejudice across both datasets (*β*_AUT_ = .30, *β*_SDO_ = .42, *β*_RAR_ = .04 in the 2012 ANES; *β*_AUT_ = .42, *β*_SDO_ = .39, *β*_RAR_ = .14 in the 2016 ANES). Including those social and ideological attitudes largely improved the model fit (χ^2^/*df* = 7.33 in the 2012 ANES and 11.38 in the 2016 ANES) but it still did not fit the data well.

**Fig 2:**
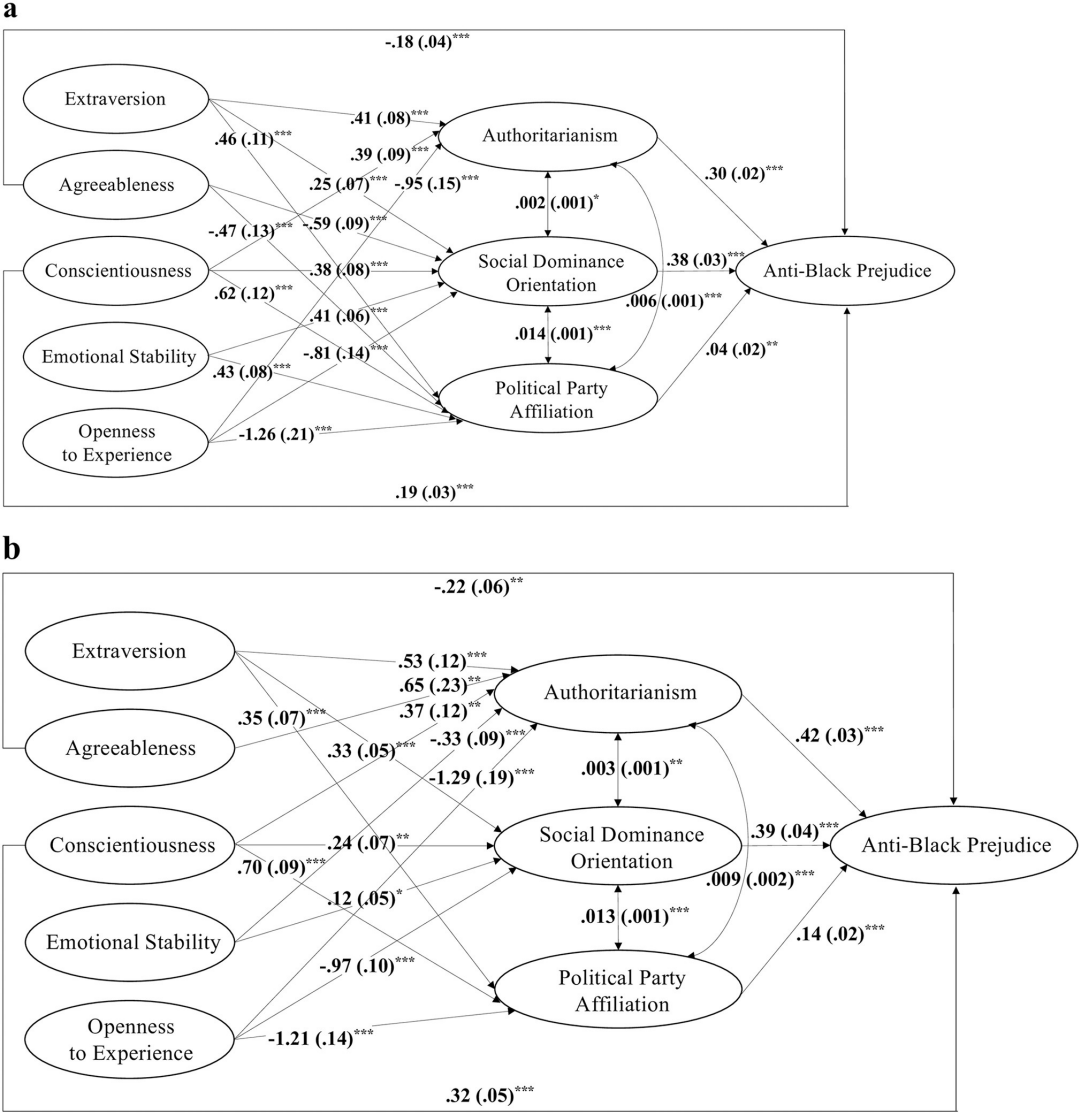
Coefficients and standard errors (in parentheses) of all significant paths (*p*-value: *** < .001, ** < .01, * < .05) in our hypothesized structural equation model estimated with applying survey weights for the 2012 ANES dataset (a) and the 2016 ANES dataset (b). All the Big Five personality traits were found to be correlated, except that between extraversion and agreeableness in the 2012 ANES dataset; to simplify the diagram, those correlations, as well as the composite indicators and the paths from the composite indicators to the latent variables were not depicted.

The third step inspected our full hypothesized model (Fig 1). Including the direct paths from agreeableness, conscientiousness, and openness to experience to anti-black prejudice substantially improved model fit (χ^2^/*df* = 2.087 in the 2012 ANES and 2.627 in the 2016 ANES). In the 2012 ANES dataset, all hypothesized paths were significant except for three: those from agreeableness and emotional stability to authoritarianism and that from openness to experience to anti-black prejudice. In the 2016 ANES dataset, four hypothesized paths were not significant: the one from agreeableness to SDO, those from agreeableness and emotional stability to party affiliation, and the one from openness to experience to anti-black prejudice. These insignificant paths were removed, and the estimates for the final models with no insignificant paths were summarized in Fig 2. These final models fitted the respective dataset very well (χ^2^/*df* = 1.892, SRMR = .005, RMSEA = .017, AGFI = .999, CFI = .999 in the 2012 ANES; χ^2^/*df* = 3.385, SRMR = .007, RMSEA = .031, AGFI = .999, CFI = .997 in the 2016 ANES). Critically, across both datasets, agreeableness and conscientiousness consistently showed significant direct associations with prejudice when authoritarianism, SDO, and party affiliation were controlled. Contrary to our hypothesis, the association between openness to experience and prejudice were fully mediated by the three social and ideological attitudes. These findings were corroborated by the conceptual replication of the DPM model (S2 Appendix) as well as the results from unweighted data (S3 Appendix).

Finally, we examined how well our hypothesized model fit the datasets compared to the two alternative models. Every model was first run with all hypothesized paths. Insignificant paths (*p* ≥ 0.05) were removed and the model was run again; these procedures were carried out recursively until all insignificant paths were eliminated from the model. Across both datasets, our hypothesized model consistently performed much better than the alternative models (Table 3).

**Table 3:** Summary of model fit indices of our hypothesized model and two alternative models for the 2012 and 2016 ANES datasets.

Model	Paths	Model Fit Indices							
		χ2	df	*p*	χ2/df	SRMR	RMSEA	AGFI	CFI
***Hypothesized***									
2012 Full	See Fig 1	4.174	2	0.124	2.087	0.003	0.019	0.999	0.999
2012 Final	R ← E, C, O	9.459	5	0.092	1.892	0.005	0.017	0.999	0.999
	S ← E, A, C, ES, O								
	PAR ← E, A, C, ES, O								
	P ← A, C, AU, S, PAR								
2016 Full	See Fig 1	5.254	2	0.072	2.627	0.003	0.026	0.999	0.999
2016 Final	R ← E, A, C, ES, O	20.308	6	0.002	3.385	0.007	0.031	0.999	0.997
	S ← E, C, ES, O								
	PAR ← E, C, O								
	P ← A, C, AU, S, PAR								
***Alternative 1***									
2012 Full	See S1 Appendix (I)	13.441	2	0.001	6.721	0.008	0.043	0.998	0.997
2012 Final	R ← E, A, ES, O, P	20.487	6	0.002	3.415	0.008	0.028	0.999	0.996
	S ← E, A, ES, O, P								
	PAR ← E, ES, O, P								
	P ← A, C, O								
2016 Full	See S1 Appendix (I)	11.795	2	0.003	5.898	0.007	0.044	0.997	0.998
2016 Final	R ← E, A, ES, O, P	92.873	12	0.000	7.739	0.020	0.052	0.997	0.981
	S ← O, P								
	PAR ← P								
	P ← A, C, O								
***Alternative 2***									
2012 Full	See S1 Appendix (II)	1319.9	9	0.000	146.65	0.087	0.216	0.966	0.667
2012 Final	R → E, A, C, ES, O, P	995.4	10	0.000	99.540	0.081	0.117	0.977	0.750
	S → E, A, O, P								
	PAR → E, A, C, ES, O								
	P ← C, O, AU, S								
2016 Full	See S1 Appendix (II)	1028.5	9	0.000	114.27	0.062	0.214	0.949	0.755
2016 Final	R → C, ES, O, P	847.2	10	0.000	84.720	0.071	0.184	0.970	0.799
	S → E, A, C, ES, O, P								
	PAR → E, A, C, ES, O								
	P ← C, O, AU, S								

Full = model with all hypothesized paths. Final = model with no insignificant paths. E = extraversion, A = agreeableness, C = conscientiousness, ES = emotional stability, O = openness to experience, AU = authoritarianism, S = SDO, PAR = political party affiliation, P = anti-black prejudice.

## Discussion

Our present research tackles the question of to what extent individual differences in prejudice might be driven by relatively flexible social and ideological attitudes versus more stable personality traits. Understanding this question is key to the conceptualization of prejudice as well as forming strategies for reducing prejudice in our society. Prior research has yet to provide a definite answer to this question (Table 1), and the accumulated knowledge was largely built on findings from convenience samples and European samples (Table 1), which highlights the need for examining this question in more diverse, representative, and large samples.

Here we investigated the relationships between the Big Five personality traits, social and ideological attitudes, and anti-black prejudice in two large probability general population samples from the United States (*N*_1_ = 3,132; *N*_2_ = 2,483). By integrating prior findings from social psychology and political science research [18,19,42,48], we argue that both motivational goals (the threat-driven *authoritarian-conservatism motivation* and the competition-driven *dominance motivation* proposed in the DPM model) and political identity underly individual differences in anti-black prejudice in the United States. Different from the postulation of the DPM model, we argue that personality traits might have direct associations with anti-black prejudice beyond those social and ideological attitudes (Fig 1).

As hypothesized, we found that agreeableness and conscientiousness were directly associated with anti-black prejudice when controlling for authoritarianism, SDO, and party affiliation (Fig 2). This finding was reproduced across two different datasets, as well as when survey weights were applied to derive estimates that were generalizable to the target population. Different from our expectation, we did not observe a direct association between openness to experience and anti-black prejudice. One plausible explanation could be that our samples of adult Americans showed a weaker correlation between openness to experience and anti-black prejudice than European and student samples [42,73], and therefore an even weaker residual association when controlling for authoritarianism, SDO, and party affiliation. The discrepancy between our findings and the postulation of the DPM model (no direct association between personality and prejudice) might be a result of multiple factors, including the unique racial dynamics in the United States, the saliency of racial issues during the collection of our datasets, larger and more representative samples used in our study, and different inventories used for measuring relevant variables.

Besides the direct association, we also found that a large part of the association between conscientiousness and anti-black prejudice were mediated through authoritarianism, SDO, and political party affiliation across both datasets (Fig 2). The association between openness to experience and anti-black prejudice were fully mediated by the three attitudinal variables across both datasets. For agreeableness, in the 2012 ANES dataset, its association with anti-black prejudice was in a large part mediated through SDO and political party affiliation; in the 2016 ANES dataset, its association with anti-black prejudice was in a large part mediated through authoritarianism, and its positive association with authoritarianism might be explained by measuring authoritarianism with childrearing values (e.g., more agreeable individuals might prefer children to have good manners and be obedient).

We employed structural equation modeling to empirically test the causal assumptions between personality traits, social and ideological attitudes, and prejudice. The significant results we found lend credibility to the corresponding causal assumptions; though our analyses could not prove the validity of those causal assumptions, given the inherent challenges of manipulating personality traits in experimental research, our findings provide helpful candidate causal pathways that could be focused on in future longitudinal studies [74].

Our findings of the direct associations between personality traits and prejudice highlight the possibility that prejudice might be a partially flexible and partially stable variable, instead of an entirely flexible attitudinal variable or an entirely predispositional variable. However, we note that there might be other social and ideological attitudes that were not included in our study would mediate those direct associations. Our findings of the mediation relationships have encouraging implications for educators and policy makers—a substantial part of prejudiced attitudes might be reduced by intervening people’s authoritarian attitudes, social dominance attitudes, and political attitudes.

The present research has several limitations. First, the ANES Time Series Study assessed each of the measure with very few items. While research has largely agreed on the validity of the short inventory TIPI for assessing the Big Five personality traits [66–68], its reliability is not guaranteed. The validity of using childrearing values to measure authoritarianism is also under debate [30,64,75]. The highly abbreviated measures of all variables might have contributed to the absence of significant associations such as that between openness to experience and anti-black prejudice. Second, the present research relied entirely on self-reported measures; whereas, Cohrs, Kämpfe-Hargrave, and Riemann (2012) has shown that peer-report data have some incremental validity compared to self-report data in predicting these measures. Finally, both the 2012 and 2016 ANES datasets were collected during presidential election periods when racial conflict was a highly salient issue; it remains an open question whether our results will generalize to other time periods under different sociopolitical contexts.

We conclude with two important future directions arising from the present research. The first is methodological: given the different results revealed from our samples than previous convenience samples, future observational studies seeking to understand the subtle and complex relationship between personality and prejudice should try to utilize sufficiently large and representative samples [76]. The second is mechanistic: the ultimate explanation for the relationship between these variables must reside in the evolutionarily-based and/or experience-based neural mechanisms of both the individual experiencing the positive or negative feeling towards members of a group, and the members of the group stimulating this feeling. For instance, there are already claims in recent research that individual differences in personal characteristics like personality can be predicted from patterns of brain activity [77,78] and that neural activations differ between perceiving in-group and out-group members [79,80]. It is not inconceivable that brain-derived measures, such as functional magnetic resonance imaging, could shed further light on the causal relationship between personality and prejudice.

## Supporting information

S1 Appendix (DOCX)

S2 Appendix (DOCX)

S3 Appendix (DOCX)
