# Supplementary material for: Personality traits are directly associated with anti-black prejudice in the United States
Source: PLoS One. 2020 Jul 1;15(7):e0235436. doi: 10.1371/journal.pone.0235436 (PMC7329088; doi:10.1371/journal.pone.0235436)
Supplement: S1 Appendix — (DOCX) [file pone.0235436.s001.docx]

**S1 Appendix**

**I. Alternative Model 1.** Instead of assuming that individual differences in social and ideological attitudes cause individual differences in prejudice, this alternative model assumes that anti-black prejudice was the causal determinant of authoritarianism, SDO, and party affiliation. All the Big Five personality traits were allowed to correlate; to simplify the diagram, these correlations, as well as the composite indicators and the paths from the composite indicators to the latent variables were not depicted in the graph.

**II. Alternative Model 2.** Instead of assuming that individual differences in personality traits cause individual differences in social and ideological attitudes, this alternative model assumes that authoritarianism, SDO, and party affiliation predisposed the individual differences in personality. All the Big Five personality traits were allowed to correlate; to simplify the diagram, these correlations, as well as the composite indicators and the paths from the composite indicators to the latent variables were not depicted in the graph.
