# Supplementary material for: Personality traits are directly associated with anti-black prejudice in the United States
Source: PLoS One. 2020 Jul 1;15(7):e0235436. doi: 10.1371/journal.pone.0235436 (PMC7329088; doi:10.1371/journal.pone.0235436)
Supplement: S2 Appendix — (DOCX) [file pone.0235436.s002.docx]

**S2 Appendix**

**I. Conceptual Replication of DPM with the 2012 ANES Dataset.** As proposed in the DPM model, attitudinal variables measuring the *authoritarian-conservatism motivation* and the *dominance motivation* were included; attitudinal variables measuring political party affiliation were not included. Coefficients and standard errors (in parentheses) of all significant paths were presented (*p*-value: ^***^ < .001, ^**^ < .01, ^*^ < .05). The model fitted the weighted data well (normed Chi-square = 1.625, SRMR = .003, RMSEA = .014, AGFI = 1.000, CFI = 1.000).

**II. Conceptual Replication of DPM with the 2016 ANES Dataset.** As proposed in the DPM model, attitudinal variables measuring the *authoritarian-conservatism motivation* and the *dominance motivation* were included; attitudinal variables measuring political party affiliation were not included. Coefficients and standard errors (in parentheses) of all significant paths were presented (*p*-value: ^***^ < .001, ^**^ < .01, ^*^ < .05). The model fitted the weighted data well (normed Chi-square = 3.135, SRMR = .004, RMSEA = .029, AGFI = .999, CFI = .999).
