# Supplementary material for: Personality traits are directly associated with anti-black prejudice in the United States
Source: PLoS One. 2020 Jul 1;15(7):e0235436. doi: 10.1371/journal.pone.0235436 (PMC7329088; doi:10.1371/journal.pone.0235436)
Supplement: S3 Appendix — (DOCX) [file pone.0235436.s003.docx]

**S3 Appendix**

**I. 2012 and 2016 ANES Unweighted Data.** Descriptive statistics of all relevant measures in the 2012 and 2016 ANES datasets calculated without applying survey weights.

|  | **E** | **A** | **C** | **ES** | **O** | **AUT** | **SDO** | **PAR** | **Prejudice** |
| --- | --- | --- | --- | --- | --- | --- | --- | --- | --- |
| 2012 ANES Unweighted | | | | | | | | | |
| E | -- |  |  |  |  |  |  |  |  |
| A | 0.01 | -- |  |  |  |  |  |  |  |
| C | 0.10^***^ | 0.22^***^ | -- |  |  |  |  |  |  |
| ES | 0.10^***^ | 0.34^***^ | 0.31^***^ | -- |  |  |  |  |  |
| O | 0.31^***^ | 0.14^***^ | 0.21^***^ | 0.22^***^ | -- |  |  |  |  |
| AUT | 0.01 | -0.02 | 0.01 | -0.06^***^ | -0.17^***^ | -- |  |  |  |
| SDO | 0.00 | -0.12^***^ | 0.06^**^ | 0.05^**^ | -0.15^***^ | 0.21^***^ | -- |  |  |
| PAR | 0.00 | -0.03 | 0.08^***^ | 0.06^**^ | -0.12^***^ | 0.26^***^ | 0.53^***^ |  |  |
| Prejudice | 0.01 | -0.10^***^ | 0.11^***^ | -0.01 | -0.11^***^ | 0.37^***^ | 0.51^***^ | 0.42^***^ | -- |
|  | | | | | | | | | |
| Mean | 0.52 | 0.70 | 0.78 | 0.66 | 0.63 | 0.37 | 0.35 | 0.51 | 0.60 |
| SD | 0.22 | 0.18 | 0.18 | 0.21 | 0.19 | 0.21 | 0.17 | 0.26 | 0.15 |
| Alpha | 0.59 | 0.39 | 0.54 | 0.63 | 0.46 | 0.61 | 0.80 | 0.85 | 0.82 |
|  | | | | | | | | | |
| 2016 ANAES Unweighted | | | | | | | | | |
| E | -- |  |  |  |  |  |  |  |  |
| A | -0.04^*^ | -- |  |  |  |  |  |  |  |
| C | 0.12^***^ | 0.28^***^ | -- |  |  |  |  |  |  |
| ES | 0.07^**^ | 0.32^***^ | 0.35^***^ | -- |  |  |  |  |  |
| O | 0.28^***^ | 0.18^***^ | 0.19^***^ | 0.16^***^ | -- |  |  |  |  |
| AUT | 0.00 | -0.02 | 0.03 | -0.04 | -0.24^***^ | -- |  |  |  |
| SDO | 0.00 | -0.12^***^ | 0.01 | 0.05^*^ | -0.24^***^ | 0.30^***^ | -- |  |  |
| PAR | 0.01 | -0.02 | 0.07^***^ | 0.06^**^ | -0.21^***^ | 0.37^***^ | 0.51^***^ |  |  |
| Prejudice | 0.05^*^ | -0.06^**^ | 0.12^***^ | 0.03 | -0.20^***^ | 0.51^***^ | 0.52^***^ | 0.56^***^ | -- |
|  |  |  |  |  |  |  |  |  |  |
| Mean | 0.54 | 0.71 | 0.79 | 0.66 | 0.67 | 0.34 | 0.29 | 0.52 | 0.57 |
| SD | 0.24 | 0.19 | 0.18 | 0.21 | 0.19 | 0.22 | 0.17 | 0.27 | 0.19 |
| Alpha | 0.61 | 0.39 | 0.54 | 0.58 | 0.44 | 0.65 | 0.70 | 0.84 | 0.84 |

*Note.* E = extraversion, A = agreeableness, C = conscientiousness, ES = emotional stability, O = openness to experience, AUT = authoritarianism, PAR = political party affiliation (higher scores for closer affiliation with the Republican party), Prejudice = anti-black prejudice. Significance code: *** *p* < 0.001, ** *p* < 0.01, * *p* < 0.05.

**II. Hypothesized Model Tested with the 2012 ANES Unweighted Data.** Coefficients and standard errors (in parentheses) of all significant paths were presented (*p*-value: ^***^ < .001, ^**^ < .01, ^*^ < .05). The model fitted the unweighted data well (normed Chi-square = 1.282, SRMR = .005, RMSEA = .009, AGFI = .996, CFI = 1.000).

**III. Hypothesized Model Tested with the 2016 ANES Unweighted Data.** Coefficients and standard errors (in parentheses) of all significant paths were presented (*p*-value: ^***^ < .001, ^**^ < .01, ^*^ < .05). The model fitted the unweighted data well (normed Chi-square = 1.912, SRMR = .007, RMSEA = .019, AGFI = .992, CFI = .999).
